# A survey of the small RNA population during far-red light-induced apical hook opening

**DOI:** 10.3389/fpls.2014.00156

**Published:** 2014-04-29

**Authors:** Ying Li, Kranthi Varala, Matthew E. Hudson

**Affiliations:** ^1^Department of Crop Sciences, University of Illinois at Urbana-ChampaignUrbana, IL, USA; ^2^Energy Biosciences Institute, University of Illinois at Urbana-ChampaignUrbana, IL, USA

**Keywords:** de-etiolation, photomorphogenesis, miRNA, functional genomics, soybeans, apical hook

## Abstract

Photomorphogenesis is a mechanism employed by plants to regulate their architecture and developmental program in response to light conditions. As they emerge into light for the first time, dark-grown seedlings employ a rapid and finely-controlled photomorphogenic signaling network. Small RNAs have increasingly been revealed to play an important role in regulating multiple aspects of plant development, by modulating the stability of mRNAs. The rapid alteration of the mRNA transcriptome is a known hallmark of the de-etiolation response, thus we investigated the small RNA transcriptome during this process in specific seedling tissues. Here we describe a survey of the small RNA expression profile in four tissues of etiolated soybean seedlings, the cotyledons, hypocotyl and the convex and concave sides of the apical hook. We also investigate how this profile responds to a 1-h far-red light treatment. Our data suggests that miRNAs show a different global profile between these tissues and treatments, suggesting a possible role for tissue- and treatment-specific expression in the differential morphology of the seedling on de-etiolation. Further evidence for the role of miRNA in light-regulated development is given by the de-etiolation responses of a hypomorphic *ago1* mutant, which displays reduced and delayed photomorphogenic responses in apical hook and cotyledon angle to far-red light.

## Introduction

Light is an essential energy source for plants. Plants have therefore evolved a sophisticated system to sense the intensity, wavelength, direction, duration, and diurnal span of environmental light and accordingly adjust their developmental plan and architecture. The response of plants to light, in addition to photosynthesis, can be divided into three categories: photomorphogenesis, phototropism, and photoperiodism (Smith, 1974). Photomorphogenesis refers to the phenomenon wherein a plant developmentally regulates its body architecture in response to a light stimulus in a non-period-sensitive, non-directional-manner (Smith, 1974; Quail, 2002). De-etiolation, also known as seedling photomorphogenesis, is a well-studied example of photomorphogenesis. A dark-grown dicotyledon seedling displays an elongated hypocotyl, unexpanded, closed cotyledons, closed apical hook, and undifferentiated chloroplasts. De-etiolation occurs after it is exposed to light, and involves an inhibition of hypocotyl elongation, opening of cotyledons and apical hook, and chloroplast maturation. This response would typically be activated at the time that a germinated seedling emerges from soil or leaf litter into sunlight. The timing of de-etiolation is thus critical to the survival of plants (Quail, 2002), and this process is influenced both by light signals and the plant's developmental program.

Photomorphogenesis is mediated by signaling pathways that are downstream of photoreceptors, among which phytochrome was the first to be characterized (Borthwick et al., 1952; Butler et al., 1959; Smith, 2000; Nagy and Schafer, 2002; Quail, 2002, 2007; Franklin and Quail, 2010). In Arabidopsis, there are five phytochromes (phyA-phyE) collectively mediating far-red light and red-light induced de-etiolation. PhyA is responsible for the de-etiolation induced by continuous far-red light (FRc), while phyB-E are mainly responsible for red-light induced de-etiolation (Sharrock and Quail, 1989; Clack et al., 1994; Devlin et al., 1998; Quail, 2002). While red light has sufficient energy to trigger photosynthesis, far-red light does not. Therefore, FR-induced and phyA-mediated de-etiolation is an ideal model system to study light signaling pathways, because a single wavelength stimulus and a single photoreceptor are involved in initializing the cascade.

A few different yet overlapping signaling mechanisms downstream of the light-activated phytochrome have been reported (Chen and Chory, 2011). Among them, one important mechanism is that phytochrome interacts with transcription factors to regulate target gene expression. Upon photoactivation, the biologically active phytochrome translocates from the cytoplasm to the nucleus and interacts with transcription factors to regulate downstream gene expression and to trigger morphological changes (Fankhauser and Chen, 2008). The mRNAs of many genes have been identified as regulated by light downstream of phytochrome using high-throughput gene expression profiling methods, for example in Arabidopsis (Quail, 2007) and in soybean (Li et al., 2011). However, another important component of the transcriptome, the small RNA, has not been studied in the context of light.

Small RNA is a class of short, regulatory non-coding RNAs of 20–30 nt (Lee et al., 1993; Hamilton and Baulcombe, 1999), which has been shown to play an essential role in regulating plant development (Chen, 2010; Axtell, 2013). A key player in small RNA function is the ARGONAUTE family protein (Carmell et al., 2002). AGO1 was the first well characterized ARGONAUTE protein (Baumberger and Baulcombe, 2005) and is known to bind with 21 nt small RNAs as part of the RNA intermediated silencing complex (RISC) and target mRNAs sequence specifically for cleavage. In a loss-of-function mutant of *ago1*, the functions of miRNA and siRNA are greatly impaired, and the mutant therefore displays severe developmental defects (Morel et al., 2002). These defects include some aspects of de-etiolation such as altered adventitious rooting and a relatively short hypocotyl in light-grown seedlings (Sorin et al., 2005), indicating a role for small RNA in light controlled developmental processes. It has also been reported that a global regulator of light responsive transcription, LONG HYPOCOTYL 5 (HY5), regulates the transcription of eight miRNA genes (Zhang et al., 2011) in response to light, in addition to many mRNAs. The global profile of small RNA expression in specific seedling tissues during de-etiolation, however, has never been systematically examined to our knowledge. We therefore investigated whether small RNAs are regulated by light during seedling photomorphogenesis by profiling the expression of small RNA in both etiolated seedlings and de-etiolating seedlings.

**Figure 1:**
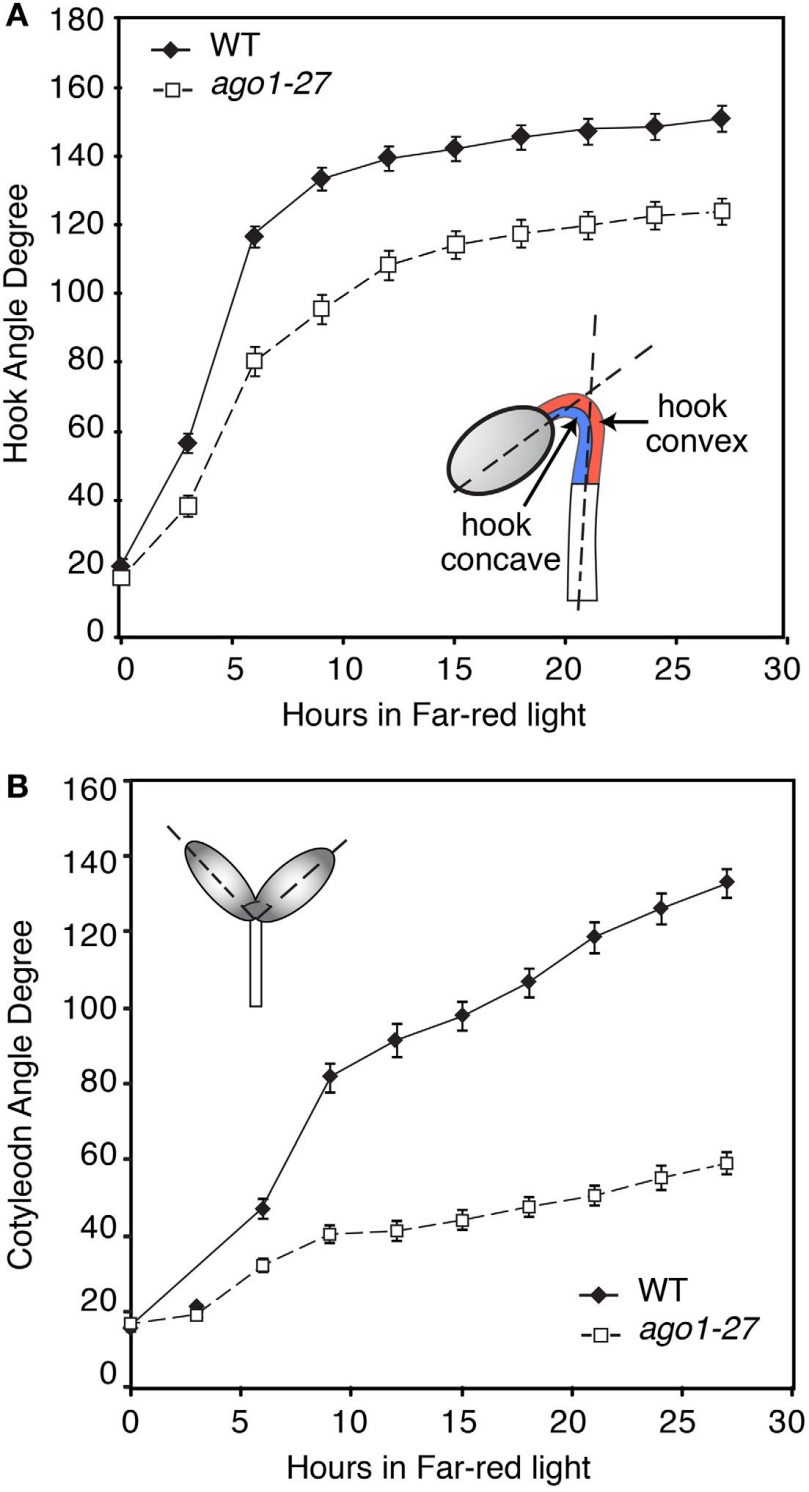
**Angle of cotyledon and hook in *ago1-27* mutants compared to background accession during growth under continuous far-red light (FRc)**. Three-day old etiolated seedlings *ago1-27* seedlings and the corresponding wild-type background accession Columbia (WT) were transferred to FRc. The angles of hook **(A)** and cotyledon **(B)** of both mutants and WT were measured from photographs taken at 3 h intervals. The error bars represent the standard error of the mean.

During seedling photomorphogenesis, distinct morphological changes and photomorphogenic gene regulation have been observed in different organs, e.g., cotyledons, hook, and hypocotyl (Li et al., 2011). Specifically, within the apical hook, the hook concave region (the half of the tissue on the inward side of the hook, see Figure 1A) and hook convex region (the outward facing half, see Figure 1A) display *opposite* morphological changes during light induced hook opening. Indeed, the light-induced changes in gene expression in the apical hook are in some cases limited to the concave region or convex region (Peck et al., 1998; Li et al., 2011). We hence examined the light regulation of small RNA in four different tissues (cotyledons, hook concave region, hook convex region, and hypocotyl) with Illumina short read sequencing. We used *Glycine max* cv. Williams 82 for this study, as soybean is an important crop in the US and, the soybean genome is completely sequenced and well annotated (Schmutz et al., 2010). The small RNA transcriptome of soybean has been recently revealed to mediate signaling and developmental events (Tuteja et al., 2008; Joshi et al., 2010; Li et al., 2012a; Hu et al., 2013). However, photomorphogenesis and the apical hook of soybean have not yet been investigated. Soybean therefore offers a good system to study the apical hook, since the miRNA transcriptome is well characterized, as is the genome, but unlike Arabidopsis, the apical hook is sufficiently large to dissect and extract sufficient small RNA for this tissue-specific type of study. Our analysis suggests that the population of small RNAs, especially miRNAs, may respond differentially in the hook to a 1-h FRc light stimulus.

## Materials and methods

### Plant material

*Glycine max* cv. Williams 82 seeds were grown in Sunshine Mix LC1 at 25°C in darkness for 7 days. Seedlings were then irradiated with far-red light (FRc) (peak 733 nm; 20 μmol m^−2^ s^−2^) for 1 h while the control seedlings were kept in darkness. Both types of seedlings were then harvested in liquid nitrogen under green safelight. For the phenotypic study of *ago1*, *ago1-27* (Morel et al., 2002) was used. The mutant genotype was verified with PCR amplification with gene-specific primers GAGGCTGTTTGCTCAGAACC and TCTACCAGCCATTCCACCTC, and then sequenced through SNP region for confirmation. The phenotyping of *ago1-27* and its background control, Columbia, were performed as previously described (Li et al., 2011).

### Illumina small RNA sequencing library preparation and data analysis

Cotyledons, hook and hypocotyls of the FRc treated soybean seedlings and dark grown seedlings were flash frozen in liquid nitrogen. The apical hooks were then transferred to RNAlater ICE (Ambion), which preserves the RNA while making the tissue more pliable for further dissection into the hook concave and hook convex without RNA change or loss. The dissection of hook concave and convex parts was done under white light with a dissection microscope, by cutting along the axis of the curved hook. For each light condition, 32 hooks were dissected this way and four pools of eight were created to make four biological replicates, so that the possible contamination caused by dissection error was minimized through the pooling process. Then, four replicate total RNA samples, each extracted from a pool of eight individual replicate seedlings, were prepared by the pine tree method (Chang et al., 1993) for each combination of tissue type and light condition. The four RNA samples of the same tissue type and light condition were then pooled in equi-molar amounts for small RNA profiling. Eight RNA samples representing the transcriptomes of cotyledons, hook convex, hook concave, and hypocotyl in seedlings after 1 h FRc treatment and dark grown seedlings were submitted to the Keck center (UIUC) for small RNA library construction and Illumina sequencing. The raw reads from Illumina sequencing were first preprocessed using FreClu with default parameters (Qu et al., 2009) to remove low-quality reads, trim adaptors, select reads with 17–35 nt size and correct for sequencing errors. In this process the read set was reduced to unique sequences with an associated abundance (counts). The preprocessed reads were then aligned to soybean tRNA and rDNA databases using novoalign while allowing up to three mismatches (www.novocraft.com). The soybean tRNA sequences were downloaded from GtRNAdb (Chan and Lowe, 2009). The soybean rDNA sequences were combined from a few resources: the NOR of soybean genome (phytozome.org, Gm13:14785374–15674994), Rfam (Gardner et al., 2008) and Gmax.rDNA.scaffolds file from phytozome V5.0. Reads mapped to tRNA or rDNA were removed from downstream analysis. Remaining reads were then mapped to the JGI soybean genome assembly (Schmutz et al., 2010) using novoalign allowing only perfect matches (*t* = 0). Reads aligning to the genome were used in the differential analysis. The unmapped reads from the last alignment (perfect matches) were then aligned to the soybean genome allowing three mismatches (*t* = 30). The reads that were still unmapped were then first aligned to the chloroplast genome and then to the mitochondrial genome (phytozome V5.0).

### Small RNA block building and counts calculation

The proximity-based small RNA block was generated as previously described (Lu et al., 2005; Li et al., 2012b). Perfectly matching reads from the eight libraries were combined to build blocks on the 20 chromosomes. The small RNA expression level of each block in a library was calculated as the sum of the abundances of small RNAs belonging to the block. A weighing method was used for calculating block counts from repetitively mapped small RNA as described in Li et al. (2012b) to prevent over-counting of the expression level of repeat-mapping small RNAs. As a result, the block counts generated with the weighted counts displayed a high degree of linearity in a pairwise comparison. By contrast, block counts generated with un-weighted small RNA counts showed a skewed x-y scatter plot and were not used for the analysis presented.

### Prediction of novel miRNA

Seventy five known *Glycine max* miRNAs and their precursors were downloaded from miRBase (as of Feb 2010). Those loci were folded with a range of parameters to determine the combination with the greatest sensitivity. By visually comparing the predicted folds from the program to the submitted folds in miRBase and scoring them individually for similarity, the following parameters were found to be optimal for finding the best secondary structure of the pri-miRNA: Folding temperature = 25°C, Threshold dG = −40 kCal/mole, Window = default, Length = 170 bp.

Goodness of the fold was determined based on the following criterion: (i) Over 75% of the bases in the small RNA need to be paired; (ii) the length of the complementary sequence (predicted miRNA*) should not be more than 1.5 times the length of the small RNA; (iii) no bases in the small RNA or within 10 bases of its end can be in the complementary strand of the stem-loop i.e., the distance between an miRNA and its complement should be at least 20 bases. Loci passing these filters were screened against known repeat sequences (GFF from http://www.phytozome.net) to remove the small number of repeat sequences that pass all these filters. These putative miRNAs were then aligned to known miRNA sequences from miRBase to identify the known miRNA and novel miRNA.

### Differential expression analysis

The following comparisons were performed: (1) For light response: FR cotyledon vs. dark cotyledons, FR hypocotyl vs. dark hypocotyl, FR hook convex vs. dark hook convex, and FR hook concave vs. dark hook concave; (2) For tissue specificity in hook: FR hook convex vs. FR hook concave and dark hook convex vs. dark hook concave. In each pairwise comparison, the Difference in Proportion method (DIP; Kal et al., 1999) was applied to select the blocks with significant difference between the two libraries. The p-values generated by DIP were corrected for multi-testing error by applying a Bonferroni correction, and any corrected *p-value* lower than 0.05 was reported to be significant. A 2-fold cutoff was also applied to select significantly regulated blocks. A low expression filter of 20 reads per million (RPM) was performed prior to the differential analysis, so that only sRNA blocks with an expression level greater than 20 RPM in at least one of the conditions in comparison are included. The DIP method features an internal normalization and, therefore a RPM normalization was not performed prior to the differential analysis.

### Annotation of FR responsive small RNA blocks

The following approaches were used to annotate the differentially expressed (DE) blocks: (i) the abundance of small RNA with different sizes in each block was computed by an in-house Perl script. (ii) the highest expressed small RNA from each block (referred to as the “key sequence” hereafter) was used to search against the miRBase mature miRNA database (Kozomara and Griffiths-Jones) using SSEARCH35 (Pearson and Lipman, 1988), to identify blocks potentially producing known miRNA (*p*-value cutoff 0.01). (iii) A strand-specific genomic sequence (170 bp) around the key sequence was tested for folding into a hairpin structure using UNAfold (Version 3.6, http://mfold.rna.albany.edu/) using parameters described above. (iv) the small RNA expression data generated in the study was visualized in Gbrowse2.0 (http://gmod.org/) with phytozome V5.0 genome annotation (http://phytozome.org/). DE blocks were classified to determine into siRNA-block-like or miRNA-block-like, by determining whether the small RNAs mapped to both strands or one strand, whether the small RNAs within the blocks mapped mostly to transposons or genes and whether it could be a likely case of cis-nat siRNA generation.

The target prediction of putative novel miRNA was done using psRNAtarget (http://plantgrn.noble.org/psRNATarget/) against Glycine max (soybean) gene models phytozome v8.0 using default parameters.

## Results

### The *ago1* mutant is impaired in photomorphogenesis

To test whether a functional miRNA system is important for photomorphogenesis, we first investigated the de-etiolation process in an Arabidopsis mutant that is defective in small RNA function. We used the hypomorphic mutant allele *ago1-27* (Morel et al., 2002), because the stronger mutant alleles (e.g., *ago1-8* and *ago1-24*) are severely defective in development and sterile, and hence are not suitable for phenotyping at the seedling stage—this also applies to other mutants we are aware of that affect the small RNA machinery. By contrast, the hypomorphogenic mutant *ago1-27*, which carries an Ala to Val mutation at amino acid 992, is nearly normal in development, and fertile. The *ago1-27* mutant and WT were grown in dark for 3 days and then given 24 h FRc treatment, during which the angles of hook opening and cotyledon opening were measured in a time series. The *ago1-27* mutants show a slower opening hook (Figure 1A) and impaired cotyledon opening (Figure 1B) in continuous FR light. This indicates that AGO1, and thus a functioning miRNA system, is required in order for Arabidopsis to de-etiolate normally. We thus conducted a profiling experiment to uncover the small RNA transcriptome during de-etiolation.

### Overview of the small RNA profile of soybean seedlings

**Figure 2:**
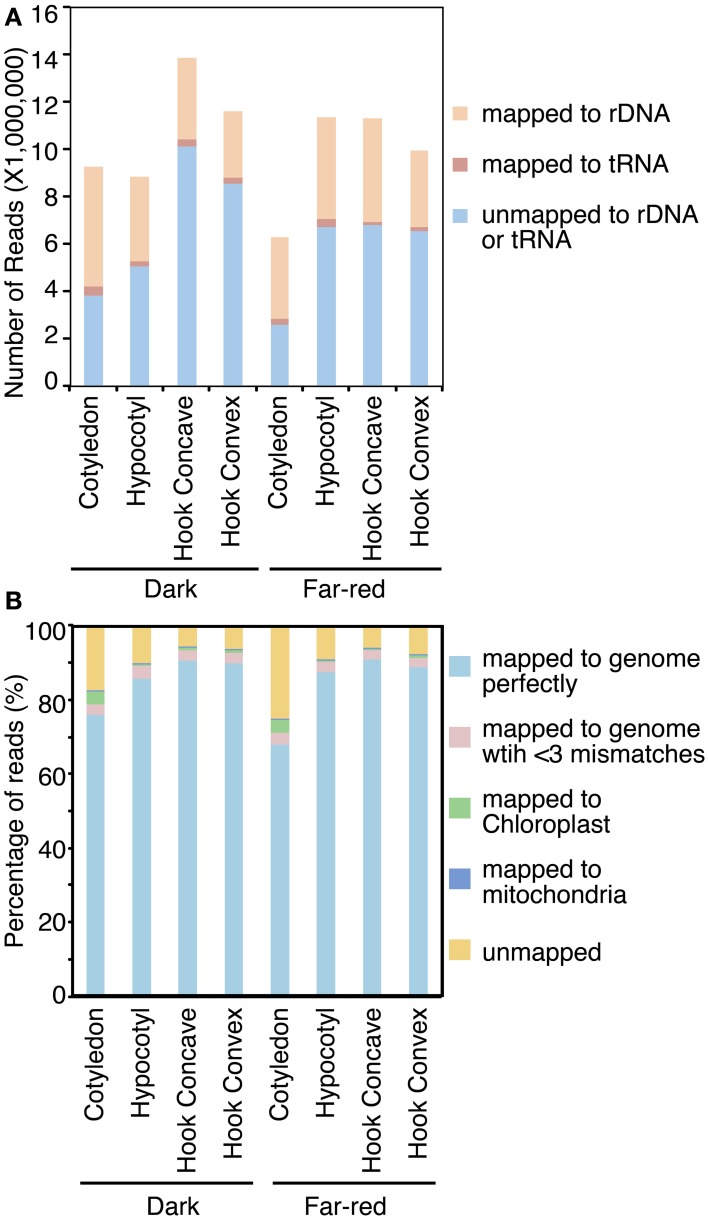
**Abundance of small RNA mapping to soybean genome**. Small RNA reads were first aligned to soybean tRNA and rDNA to filter these types of noncoding RNA **(A)**. Reads which passed this filter were then aligned to the soybean nuclear and plastid genomes **(B)**.

Total RNA samples extracted from cotyledons, the apical hook concave region, apical hook convex region and hypocotyl of 7-day-old dark-grown seedlings and seedlings treated with 1 h FRc were submitted for small RNA sequencing using the Illumina GAIIx (Keck center, University of Illinois). Eight to fifteen million reads were generated from each library (Figure 2A; Table S1). The raw reads were preprocessed and first aligned to the soybean tRNA and rDNA databases using Novoalign (www.novocraft.com). Twenty five to Fifty five percent of total small RNA reads from these libraries mapped to tRNA or rDNA databases (Figure 2A; Table S1). The two cotyledon libraries have a relatively higher percentage (approximately 55% of the total reads) of small RNA reads mapped to rDNA (Figure 2A). This higher proportion might be a result of the cotyledon samples including actively dividing young tissues, which were previously reported to produce more rDNA-mapped small RNA compared to mature tissue (Lu et al., 2005). These rDNA and tRNA mapped reads were considered unlikely to be relevant to light-responsive small RNA regulation and were hence removed from further analysis. The remaining reads were aligned to the JGI soybean genome assembly (v5.0) using Novoalign, allowing only perfect matches (*t* = 0). From 68 to 91% of the reads mapped to the soybean genome perfectly (Figure 2B). Allowing two mismatches in each read increased the percentage of mapped reads by 2.3–3.6% (Figure 2B). This is likely caused by sequencing errors, or by polymorphisms within the Williams 82 population (Varala et al., 2011). A small portion of the remaining non-mapped reads aligned to the soybean chloroplast and mitochondrial sequences (Figure 2B). Finally, 6.0–25.4% of reads remained unmapped to either the nuclear genome or the plastid genome even with relaxed stringency (Figure 2B). In summary, most reads mapped to the soybean nuclear genome assembly perfectly, resulting in 1.7–9 million nuclear genome-mapped reads representing 0.5–2.5 million unique sequences for each library. Those reads were used for further analysis (Table S1).

**Figure 3:**
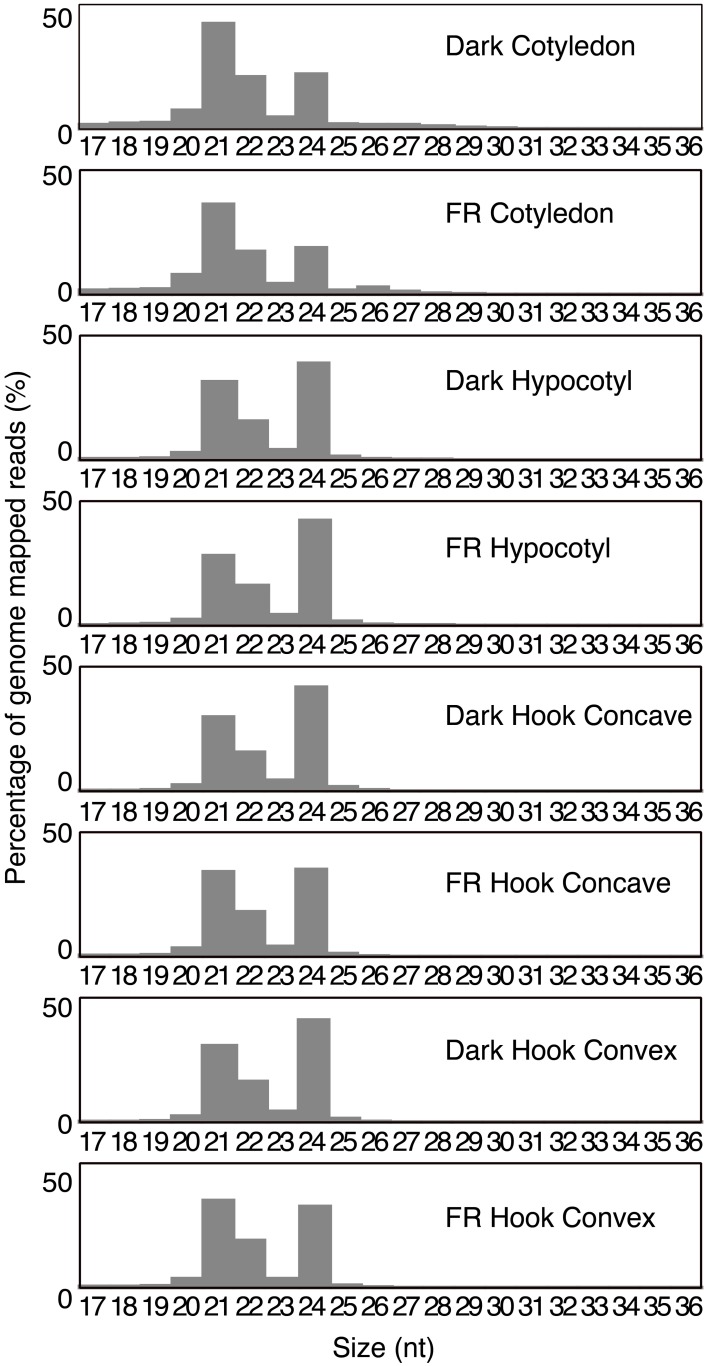
**Abundance of size classes of small RNA (17–36 nt)**. The percentages of the abundance of genome-mapped small RNA of each size class are plotted for each library.

Different functional categories of small RNA are known to have specific sizes (Chen, 2009, 2010; Axtell, 2013), thus we analyzed the size distribution of the genome-mapped small RNAs. The 21 and 24 nt small RNAs are the two most abundant classes in all libraries (Figure 3). Interestingly, a distinctive 21:24 nt ratio was observed across different tissue types. The 21 nt small RNAs represent the most abundant class in the cotyledons, with a 21:24 nt ratio close to 2:1 in both libraries, the highest of the tissues examined. In contrast, the 24 nt class is the most abundant in both hypocotyl libraries, with 21:24 nt ratios of 0.81:1 and 0.67:1, respectively. Among the four hook libraries, the dark treated plants resembled the hypocotyl libraries, with the 24 nt class as the most abundant class (21:24 nt ratio of 0.71:1 and 0.75:1 for hook concave and hook convex, respectively), however, surprisingly, the FR libraries had almost equal amounts of 21 and 24 nt small RNA (21:24 nt ratio as 0.98:1 and 1.07:1). This may indicate either an induction of 21 nt class or a repression of 24 nt sRNA in response to FR. Most miRNAs belong to the 21 nt class, while the 24 nt class mainly consists of heterochromatic siRNA (Chen, 2010). Therefore, the change in ratio of 21:24 nt may indicate that miRNAs are relatively more abundant in response to FR treatment in the hook within 1 h. The proportion of 22 nt small RNA, reported to be ta-siRNA triggers (Chen, 2010), is relatively constant across the eight libraries (Figure 3).

### Identification of novel miRNAs in de-etiolating soybean seedlings

To test whether there are novel miRNAs in our dataset, a pipeline was developed to identify putative novel miRNAs based on the secondary structure of the putative pri-miRNA as well as all other standards considered necessary for description of a new miRNA (Meyers et al., 2008). We included small RNA data from a global profile of small RNAs in soybean to broaden the scope of this analysis to cover a wide range of organs and developmental stages. Specifically, data from our experiment was combined with data from Zabala et al. (2012) to create a broad profile of endogenous small RNA populations in soybean. Appropriate parameters for maximizing the sensitivity of the prediction process were determined using a training set of known miRNA generating loci in Glycine max in miRBase (mirbase.org; Kozomara and Griffiths-Jones, 2011). Combinations of parameters, including folding temperature, threshold dG, and the length of flanking sequence were tested using UNAfold (Markham and Zuker, 2008). The learned set of parameters was then used to select small RNA producing loci, from the combined dataset, that show a favorable secondary structure.

In soybean, identical miRNAs are generated by multiple loci distributed across the genome. For example, the highly conserved miRNA miR156 is encoded by 12 independent loci. Accordingly, small RNAs from the soybean libraries that mapped to less than or equal to 20 genomic loci were submitted to the prediction pipeline. Approximately 200,000 loci were tested within this set and after a few filtering steps (see Methods), a total of 188 putative novel miRNA sequences were identified (Table S10). These predicted miRNAs include 21, 22, and 24 nt long representative sequences. For further analysis, only the predicted miRNAs from the 20–22 nt classes (137 out of the 188) were chosen for further studies (Table S2).

**Figure 4:**
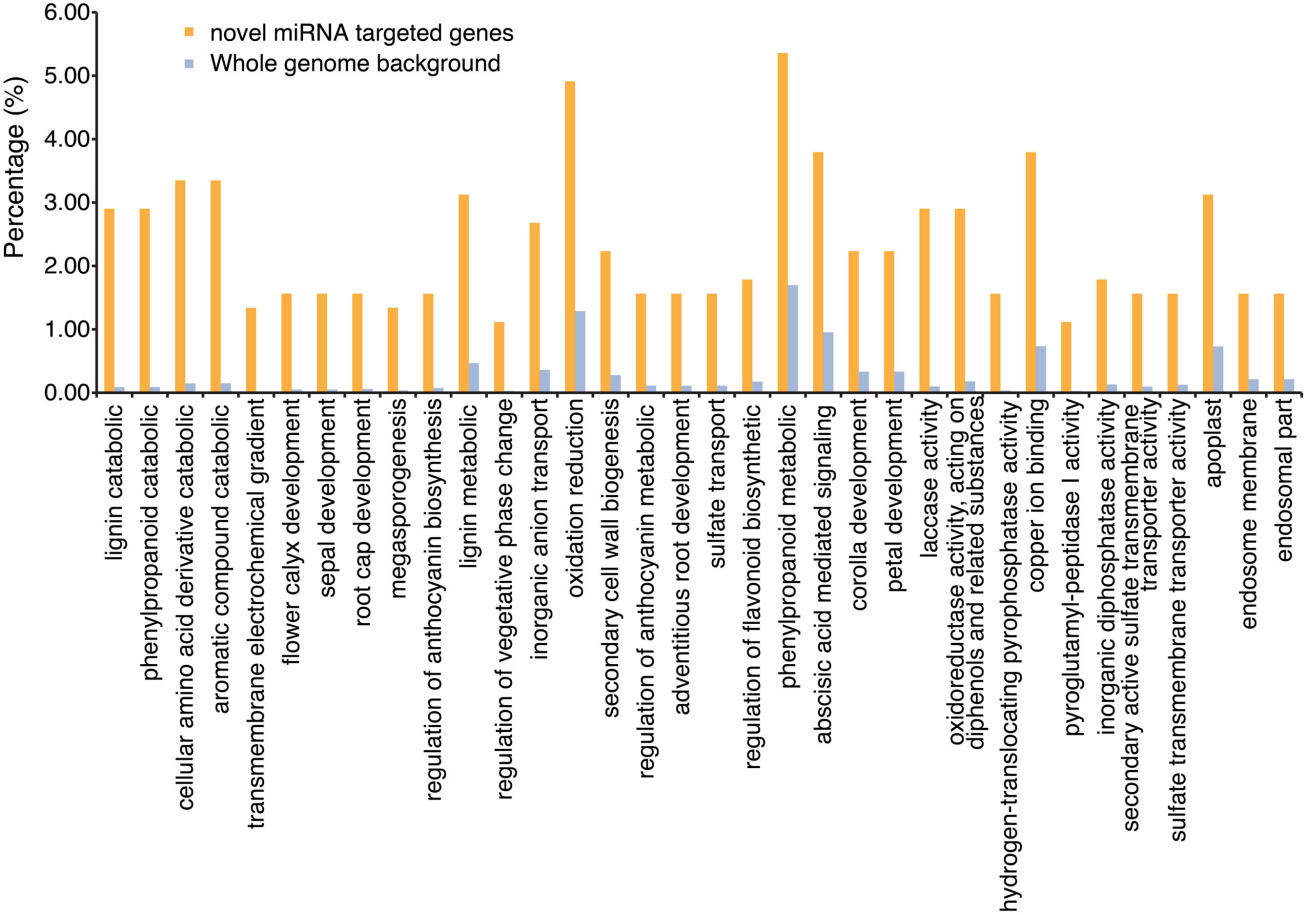
**Over-represented Gene Ontology terms associated with predicted mRNA targets of the putative novel miRNAs found in this study**. Gene Ontology (GO) terms associated with these gene models were processed to identify over-represented GO terms, using AgriGO (bioinfo.cau.edu.cn/agriGO). The graph indicates the percentage of predicted target mRNAs associated with a particular term that is significantly over-represented, and the percentage of all mRNAs in the genome associated with the same term. Significantly over-represented GO terms were determined by hypergeometric test with an FDR correction (alpha = 0.05).

An investigation of the role these miRNAs might play in soybean transcriptional control was performed by computationally identifying the potential targets of these miRNAs. psRNATarget (Dai and Zhao, 2011) was used to identify the potential targets of the predicted novel miRNAs. With the exception of two miRNA sequences, all novel miRNAs were predicted to target multiple genes in the soybean genome (Table S3). A total of 1094 gene models in the soybean genome were identified as potential targets with a large majority predicted to be targeted for cleavage, as opposed to translational regulation (Table S3). Gene Ontology (GO) terms associated with these gene models were processed to identify over-represented GO terms, using AgriGO (bioinfo.cau.edu.cn/agriGO). Significantly over-represented GO terms were determined by a t-test, assuming a hypergeometric distribution, and an FDR correction at alpha of 0.05 (Figure 4; Table S4).

### FRc-responsive small RNA clusters

When mapping small RNAs to the genome, siRNAs sharing the same targets and/or biogenesis are usually found in a dense cluster on both strands of a siRNA-generating genomic locus (e.g., a transposon or a targeted protein coding gene); in contrast, a miRNA usually co-localizes with an miRNA* on the transcribed strand of the miRNA gene. When comparing between two libraries, individual siRNA may be different between two libraries, while the overall counts of small RNA from an siRNA generating genomic locus (which is more biologically relevant) do not change. To capture the biologically relevant small RNA regulation, a proximity-based clustering algorithm was used to build 309,645 blocks on the genome from a pool of small RNA reads from all libraries. The mean length of the blocks is 2621 bp and the median 575 bp. Each block represents a genomic region where potentially functionally related small RNAs are generated. The abundance of small RNAs in a block was summed to represent the small RNA expression level of the block in a library (hereafter referred to as block count).

The block counts from the dark-grown libraries were compared with the FRc-treated libraries using the DIP statistical test to identify FRc-responsive DE blocks in the cotyledons, hook convex region, hook concave region, and hypocotyl. The p-value computed by DIP was corrected with Bonferroni multi-testing control (Kal et al., 1999). We counted DE blocks that had a corrected p-value smaller than 0.05, and we also imposed a fold change cutoff of FR/dark or dark/FR greater than 2-fold. With these criteria, 11 blocks were identified as being regulated by FRc in the cotyledons, 57 in the hook concave side, 139 in hook convex side, and 25 in hypocotyl (Table S5). The small RNA expression profiles of the hook concave region were also compared to those of the hook convex region under both light conditions, leading to the identification of 4 blocks DE between the hook convex and hook concave in the darkness, and 26 blocks after 1 h FRc treatment (Table S5).

### Functional annotation of the FRc responsive small RNA blocks

**Figure 5:**
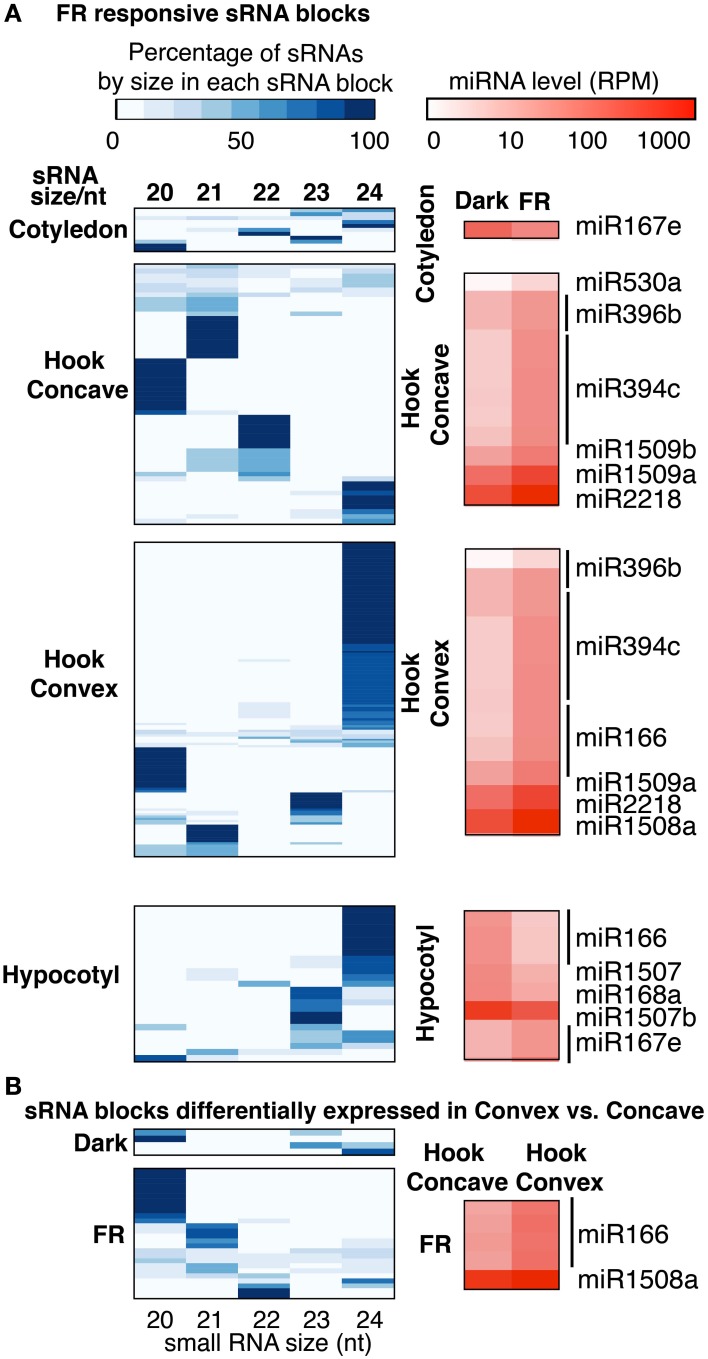
**(A)** Size distribution of sRNAs and expression level of miRNAs in FR responsive sRNA blocks and **(B)** size distribution of sRNAs and expression level of miRNAs for sRNA blocks differentially expressed in the concave vs. convex side of the apical hook. The percentages of the total abundance of each small RNA species with lengths 20–24 nt in differentially expressed blocks are shown in the white-blue heatmaps. The log10 expression levels of previously characterized miRNAs are shown in the white-red heatmaps.

To identify miRNA or siRNA associated with the FR-responsive small RNA blocks, we investigated the DE blocks with multiple approaches to characterize their biological functions. First, we examined the length distribution of small RNAs in each DE block, since different categories of small RNAs have different sizes (Axtell, 2013). For example, most microRNAs are 21 nt in length, and the 23/24 nt small RNAs usually represent heterochromatic siRNAs. Therefore, the abundance of small RNA from 20 to 24 nt in each DE block was plotted by length (Figure 5). Most of the DE blocks identified as DE in hypocotyl in response to FR are dominated by 23/24 nt small RNA (Figure 5A), while DE blocks identified from the comparison of the hook concave region and hook convex region after 1 h FRc are dominated by 20/21/22 nt small RNA (Figure 5B). This result again suggested that miRNAs are actively regulated in response to FR treatment in the hook within 1 h, supporting our previous result. DE blocks identified from the other comparisons have comparable numbers of blocks dominated by different size classes (Figure 5).

To identify known miRNA genes in the DE blocks, the most highly expressed small RNA from each block (hereafter termed the “key sequence”) was used for a sequence similarity search in miRBase (mirbase.org; Kozomara and Griffiths-Jones, 2011). Meanwhile, to identify both known and novel miRNA genes in the DE block, the genomic sequence surrounding the key sequence was extracted to test whether it could fold into a canonical stem-loop structure commonly observed in pri-miRNA. Additionally, the small RNA expression profile across the block was visualized using Gbrowse v.2 to determine whether the block resembled the expected features of a miRNA generating locus (i.e., production of 21/22 nt RNA predominantly from two sites: the miRNA and miRNA*). If a block fails to satisfy the above criteria, it is likely to be a siRNA generating block, in which case it was recorded whether most of the siRNAs in the block map to a protein-coding gene or a transposon according to the phytozome soybean genome annotation (v.5). Using the pipelines described above, the DE blocks were annotated as miRNA loci, siRNA blocks matching protein-coding genes or siRNA blocks matching transposable elements (TE). The results for miRNA blocks and siRNA blocks are discussed separately below.

### Tissue-specific FRc-responsive miRNA

If (1) a foldback structure meeting our criteria could be identified from a DE block, (2) the key sequence matched a known miRNA, (3) the predominant small RNA class is 20/21/22 nt, and (4) the small RNA expression profiles of the block displays the features of a miRNA gene locus described previously (sparse cluster dominated by a 20–22 nt sequence miRNA and miRNA*), the block was identified as a known miRNA gene.

**Figure 6:**
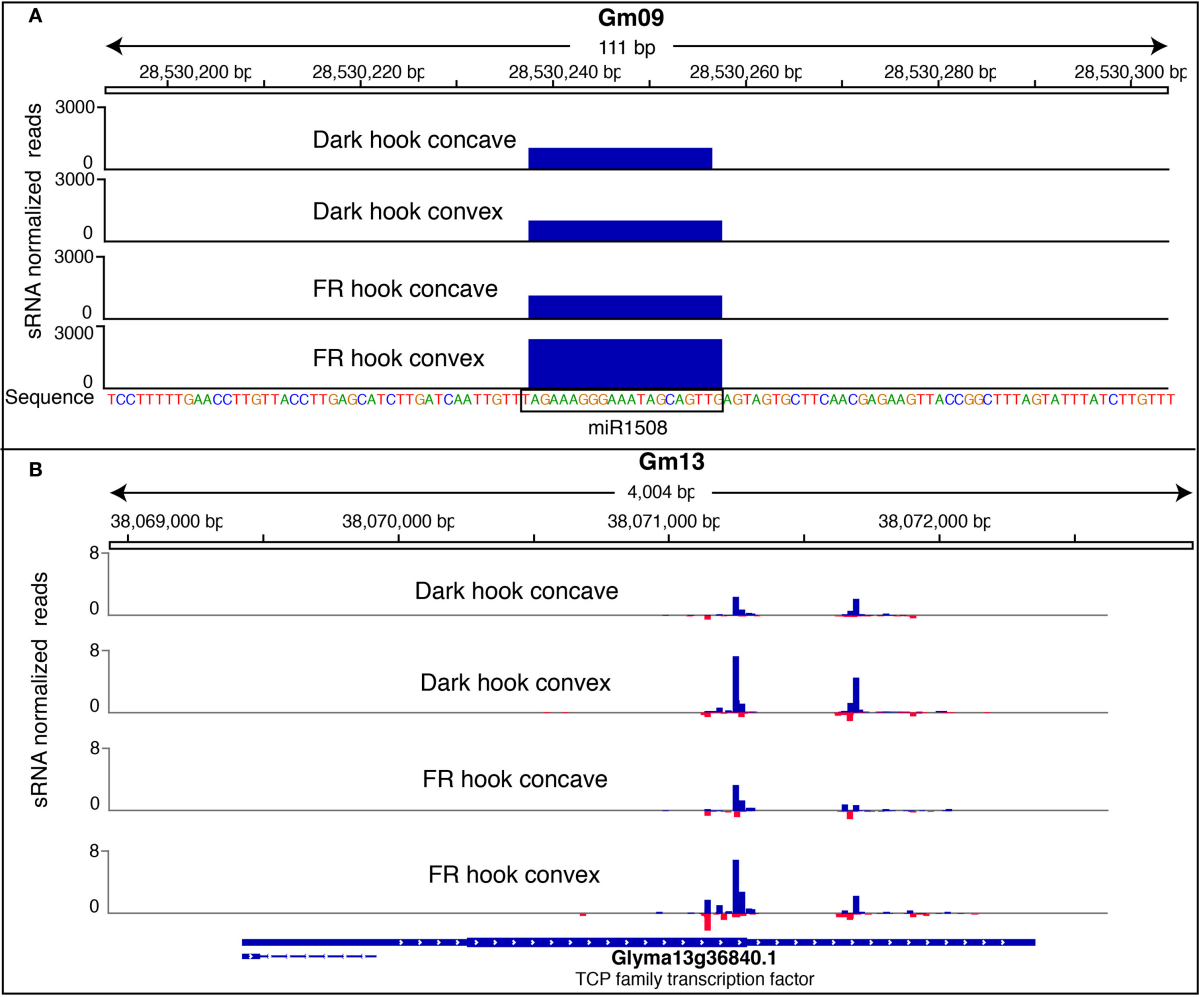
**Graphs showing the small RNA reads mapping to a miRNA block (A) and siRNA block (B). (A)** A hook-convex specific FRc-responsive miRNA miR1508 was shown as an example. The mature miR1508 reads was mapped to a genomic region in Gm09. Each sample was represented as a track, while the Y-axis of the track represents the RPM normalized weighted counts of the sRNA reads. **(B)** A siRNA block with higher expression level in hook convex than hook concave co-localizes with a protein-coding gene Glyma13g36840. Each sample was represented as a track, while the Y-axis of the track represents the RPM normalized weighted counts of the sRNA reads (negative value means mapping to reverse strand). Blue bar represents sRNA mapping to the forward strand, and red bar represents sRNA mapping to the reverse strand. The graph was prepared with Integrative Genomics Viewer (Thorvaldsdóttir et al., 2012).

Using these criteria, we found several miRNAs that met our definition of being potentially DE in response to FRc in cotyledons, the hook concave region, hook convex region or hypocotyl. miR167 was down-regulated by FRc in the cotyledons (Figure 5A). miR394, miR396, miR530, miR1509, and miR2218 were up-regulated by FRc in the hook concave region (Figure 5A), while miR166, miR394, miR396, miR1508, miR1509, and miR2218 were up-regulated by FRc in the hook convex region (Figure 5A). Collectively, this suggests that miR394, miR396, miR1509, and miR2218 were up-regulated in the apical hook by FRc; In the hypocotyl, miR168, miR166, and miR1507 were down regulated and miR167 was up-regulated by FRc (Figure 5A; Table S6). Interestingly, the comparison between the hook convex region and hook concave region of FRc treated seedlings showed that miR166 and miR1508 were expressed higher in the hook convex region than the hook concave region under FRc (Figure 5B; Table S7). Therefore, miR166 and miR1508 are likely induced by FRc only in the hook convex (Figure 6A). For some of the microRNAs, e.g., miR166, miR167, and miR396, their mRNA targets have been well characterized to regulate plant growth and development (Williams et al., 2005; Wu et al., 2006; Jung and Park, 2007; Liu et al., 2009; Rodriguez et al., 2010). For the other identified FR-responsive miRNA, little is known about their mRNA targets. We used psRNAtarget (Dai and Zhao, 2011) to predict the potential targets of those miRNAs (Table S8). None of the predicted novel miRNAs that we identified in this study (Table S2) was detected to be regulated by FRc.

### siRNA blocks

If a DE block does not have a key sequence similar to a known miRNA and does not form a stem-loop foldback structure, it likely represents a siRNA-generating locus. We visualized the small RNA expression of those blocks in Gbrowse v2.0 to confirm that they showed a similar pattern to that expected for siRNA generating loci (i.e., generation of many different small RNA species across the block). We then investigated if the majority of siRNAs in those blocks map to any gene or TE, based on the phytozome (www.phytozome.org) annotation (v5.0) of the soybean genome. As a result, 7 of the 57 FR responsive blocks in the hook concave sample and 36 of the 139 FR responsive blocks in the hook convex sample featured siRNA mapping to TEs. Interestingly, all of these are down-regulated by FRc. These small RNAs are mostly 24 nt, and are likely heterochromatic siRNAs repressing transponson activity to maintain genome integrity. No DE block with TE-mapping small RNA was identified among the FR responsive blocks identified in the cotyledons and hypocotyl. In 24 DE blocks from various tissues, the majority of small RNAs mapped to a high confidence gene, suggesting that many of the small RNAs identified may regulate gene expression, either in response to light or differentially in the hook convex region compared to the hook concave region (Table S9). The functions of those genes were annotated using a homology search against the model organism Arabidopsis (Table S9). One such gene Glyma13g36840, which encodes a TCP family transcription factor, has more mapped siRNA reads in the hook convex compared to hook concave, and was shown as example in Figure 6B.

## Discussion

The ability to respond to environmental light allows plants to adapt rapidly to environmental changes. It has been known for a long time that the plant light responses involve induction and repression of protein-coding gene expression at the mRNA level. Recently, small RNA has been implicated in plant development and abiotic stress responses (Lewis et al., 2009; Chen et al., 2010). However, the role of small RNA in light-related plant development has not been investigated to our knowledge. In our study, we showed that a mutant with defective miRNA function displayed impaired de-etiolation. We further performed a survey sequencing of the small RNAs in the cotyledons, hook convex region, hook concave region, and hypocotyl, both from etiolated seedlings and seedlings treated with 1-h FRc light. We showed that potentially novel miRNAs may exist in these little-characterized tissues, and that the level of some small RNAs met our statistical criteria for differential expression after a 1-h light treatment in the specific regions of the seedling investigated. Together these results expand our knowledge of the role of small RNA in plant development, as well as add to our current understanding of light signaling.

First, by developing a novel miRNA prediction pipeline when the data was first available, we identified 137 putative miRNA that are novel to the miRBase at that time (2010). To compare our predicted miRNAs with more recent studies on miRNA identification, we compared the 137 novel miRNAs with a current miRBase database at the time of writing (March 2014), and found that 73 of our previously predicted novel miRNAs have been reported in other studies as novel miRNAs (Table S2). This result underscores the consistency of our novel miRNA prediction pipeline with those used by other researchers. Interestingly, one of the miRNAs recently added to miRBase, miR395 (CUGAAGUGUUUGGGGGAACU), is predicted by our analysis to target Glyma11g36210, which is rapidly reduced in expression in response to FRc in the soybean apical hook as reported in Li et al. (2011). Glyma11g36210 encodes a sulfate transporter, SULTR2;1. Thus not only is a sulfate transporter rapidly down-regulated by light in the apical hook, but this rapid down-regulation is likely facilitated by miRNA-mediated turnover of the transcript.

A major advantage of using soybean as the experimental system to study hook opening is that, compared to the model organism Arabidopsis, the soybean hook is large and relatively robust, and thus relatively easy to dissect into the oppositely-responding convex and concave regions. In our study, the dissection of concave vs. convex regions was facilitated by a novel method of using a fixative on flash-frozen apical hook tissue (RNAlater ICE), which preserves the RNA and prevents biochemical responses to dissection or light, while making the tissue soft for dissection at low temperatures. Therefore, by dissecting along the middle axis of the curved hook under a binocular microscope under white light, the RNA expression patterns can be attributed to the responses that occurred before freezing and fixation. This process both addresses concerns that light or wounding would cause a response in RNA expression, and that the dissection process may cause variable RNA degradation, for example by means of releasing degradative enzymes from lytic compartments. For each light condition examined experimentally, 32 hooks were dissected in this manner and pooled for each RNA sample used for library generation. The possible contamination caused by errors in the dissection boundary is minimized by this pooling process.

In our study, the proportion of 21-nt small RNA in the hook convex and hook concave regions increased greatly after a 1 h FRc treatment (Figure 3). Since most identified miRNAs in plants belong to the 20/21/22 nt class, this likely suggests that the overall level of miRNA is up regulated by light in the hook region. While it is also true that siRNA can be generated in a 21 nt form, the identification of up-regulated miRNAs in the hook provides evidence to support this (Figure 5A). Combined with the fact that the hypomorphic *ago1* mutant, expected to slow but not eliminate miRNA directed cleavage, shows a slower-opening hook and cotyledons during de-etiolation, we propose that light induces expression of miRNAs in the hook region, and this triggers mRNA degradation required for normal hook opening and subsequent cotyledon opening.

A few known miRNAs identified here as light responsive have well characterized functions. miR167, for example, is known to target *Auxin Response Factors ARF6* and *ARF8* to correct the patterning of *ARF6* and *ARF8* expression domains during ovule and anther development (Wu et al., 2006). Recently, soybean miR167 was also implicated in Nematode defense (Li et al., 2012a). In our study, miR167 was found to be DE. While this expression pattern remains to be confirmed by another technique, such as qPCR or a Northern blot, our data indicates it is down regulated in the cotyledons and up regulated in the hypocotyl in response to a 1 h FR stimulus. This is consistent with changes in cellular expansion in these organs, with an active expansion of the cotyledons and an inhibition of growth in the hypocotyl during de-etiolation. miR396 is also a well characterized and well conserved miRNA. It targets *Growth Regulating Factor* (GRF) transcription factors, which regulate leaf growth (Liu et al., 2009; Rodriguez et al., 2010). The miR396 level was at higher levels in response to FRc in both sides of the hook in our study, again requiring confirmation, but possibly part of a mechanism to alter growth regulation. Our data also suggested, without confirmation, that two miRNAs, miR1508, and miR166, are up-regulated by FRc specifically in the hook convex region. In Arabidopsis, miR166 targets *HD-ZIPIII* genes which regulate many aspects of plant development, including shoot apical meristem architecture (Williams et al., 2005; Jung and Park, 2007), vascular patterning (Zhong and Ye, 2007), leaf polarity (Juarez et al., 2004; Nogueiral et al., 2007), and floral development (Jung and Park, 2007). Recently, miR166 was implicated in soybean Nematode defense (Li et al., 2012a). An induction of miR166 in response to FRc in the hook convex in our study may suggest miR166 and its target *HD-ZIPIII* genes are involved in the opening of apical hook. Since the global mRNA response to FRc in cotyledon, hook and hypocotyl in soybean was examined in Li et al. (2011), we crosschecked the targets of FR-responsive miRNA and siRNA identified in this study with the results of Li et al. (2011), but no overlap was found. However, since both studies focus on changes in expression within 1 h of exposure to light, it is unlikely an miRNA change in expression will have a two-fold effect on a mRNA within this time period. Therefore, replicated time series data will likely be necessary to capture the negative correlation between FR-responsive sRNA and FR-responsive mRNA.

In conclusion, the miRNA mechanism (specifically the AGO1 dependent miRNA function*)* is needed for the normal, rapid responses of seedlings to FR. In conjunction with this result, the profile of miRNA expression is noticeably different between seedling tissues, between the concave and convex sides of the apical hook, and between dark-grown and FR-treated seedlings. These changes include miRNAs known to regulate cell expansion and organ growth. While further confirmatory experiments are necessary to verify the responses of individual miRNAs described here, at least one of the profile changes fits with changes in expression of a potential miRNA target described in another study. Together these findings suggest that the miRNA system is implicated in regulating the rapid morphological responses to light in the apical region of dicot seedlings.

## Author contributions

Matthew E. Hudson and Ying Li designed the experiment. Ying Li performed the phenotypic analysis, tissue culture, and RNA extraction. Ying Li and Kranthi Varala performed the data analysis. Ying Li, Kranthi Varala, and Matthew E. Hudson prepared the manuscript. Ying Li and Kranthi Varala prepared the figures and tables.

## Conflict of interest statement

The authors declare that the research was conducted in the absence of any commercial or financial relationships that could be construed as a potential conflict of interest.
